# Multi-photon ionisation spectroscopy for rotational state preparation of ${{\bf{N}}}_{{\bf{2}}}^{{\boldsymbol{+}}}$

**DOI:** 10.1038/s41598-018-36783-5

**Published:** 2019-01-24

**Authors:** Amy Gardner, Timothy Softley, Matthias Keller

**Affiliations:** 1 ITCM Group, Department of Physics and Astronomy, University of Sussex, Falmer, BN1 9QH United Kingdom; 2 University of Birmingham, Edgbaston, Birmingham, B15 2TT United Kingdom

## Abstract

In this paper we investigate the 2 + 1′ resonance enhanced multi-photon ionisation (REMPI) of molecular nitrogen via the *a*^1^Π_*g*_(*v* = 6) intermediate state and analyse its feasibility to generate molecular nitrogen ions in a well defined ro-vibrational state. This is an important tool for high precision experiments based on trapped molecular ions, and is crucial for studying the time variation of the fundamental constant *m*_*p*_/*m*_*e*_ using ${{\rm{N}}}_{2}^{+}$. The transition is not reported in the literature and detailed spectral analysis has been conducted to extract the molecular constants of the intermediate state. By carefully choosing the intermediate ro-vibrational state, the ionisation laser wavelength and controlling the excitation laser pulse energy, unwanted formation of rotationally excited molecular ions can be suppressed and ro-vibrational ground state ions can be generated with high purity.

## Introduction

High precision experiments with molecules span over many research areas; from ultra-cold chemistry^1–3^ to proposals for quantum computing^4^. Additionally high resolution spectroscopic studies with molecules have motivations for improved frequency standards^5,6^, testing fundamental theories^7–9^, and searching for changes in fundamental constants^10,11^.

Molecular ions in traps provide a good isolated environment for such experiments, benefiting from long trapping lifetimes and good spatial localisation. As well as this multiple species can be trapped within a single potential, with the shared motional modes opening up new realms of experimental control^12^. Cooling molecular ions to the motional ground state has recently been demonstrated^13,14^, and it is also possible to transfer state information between species trapped under these conditions^15–18^. Fundamental to such experiments is the ability to successfully prepare the internal states of the molecular ions^19^. Various techniques have been used depending on the particular requirements posed by the species, for instance laser cooling has been demonstrated for heteronuclear diatomics^20–22^. In these cases, the strong interaction of the polar molecules with blackbody radiation (BBR) aids the cooling scheme by redistribution of the population. Techniques such as buffer gas cooling^23^ and use of hybrid traps^24,25^ can also be used to reduce the internal temperatures of trapped ions.

Another state-preparation technique is to use the ionisation process itself to generate molecular ions in a specific internal state. By setting the ionisation energy used to just above the threshold of the desired state, it is possible to load ions with a high rotational state purity. Resonance enhanced multi-photon ionisation (REMPI) techniques have traditionally been used for the investigation of the internal structure of molecules^26–28^, however its use has also been demonstrated for state preparation in ion traps for a number of different species. A molecular ion of particular interest for high precision spectroscopy experiments is ${{\rm{N}}}_{2}^{+}$. Transitions in nitrogen ions have been proposed as very suitable candidates for investigating the time variation of the fundamental constant *μ*, the mass ratio of the proton to the electron^29^. As well as having a high sensitivity to *μ*, ${{\rm{N}}}_{2}^{+}$ does not suffer from large systematic effects, therefore fractional systematic frequency uncertainties can be below 10^−18^. Due to its apolar nature and its high ionisation energy, ${{\rm{N}}}_{2}^{+}$ cannot be prepared in its internal ground state via buffer gas collisions nor is it accessible for blackbody radiation assisted laser cooling. Therefore, it must be prepared in its rovibrational state in the ionisation process.

Rotationally state selected ${{\rm{N}}}_{2}^{+}$ has been formed in ion traps previously, using a 2 + 1′ REMPI scheme via the intermediate $a{^{\prime\prime} }^{1}{{\rm{\Sigma }}}_{g}^{+}({\rm{\nu }}=\mathrm{0)}$ state^30,31^. Ions were successfully loaded in their rovibrational ground-state with a stated efficiency of 93 ± 11%. However, a drawback to this scheme is that the excitation photons have a higher energy than the final ionisation photon. This means that there is a strong contribution from a single colour 2 + 1 REMPI process with the excitation laser alone, and so this laser’s fluence needs to be reduced, resulting in a reduction of the overall ionisation efficiency. In this paper we investigate a novel 2 + 1′ REMPI scheme via the ${a}^{1}{{\rm{\Pi }}}_{g}({\rm{\nu }}=\mathrm{6)}$ intermediate state. With a two-photon resonance at 39270 cm^−1^ and the final ionisation step at 47110 cm^−1^, this scheme prevents single colour 2 + 1 REMPI processes and thus allows state selective ionisation with high laser fluence.

## Results

### 2 + 1′ REMPI

**Figure 1:**
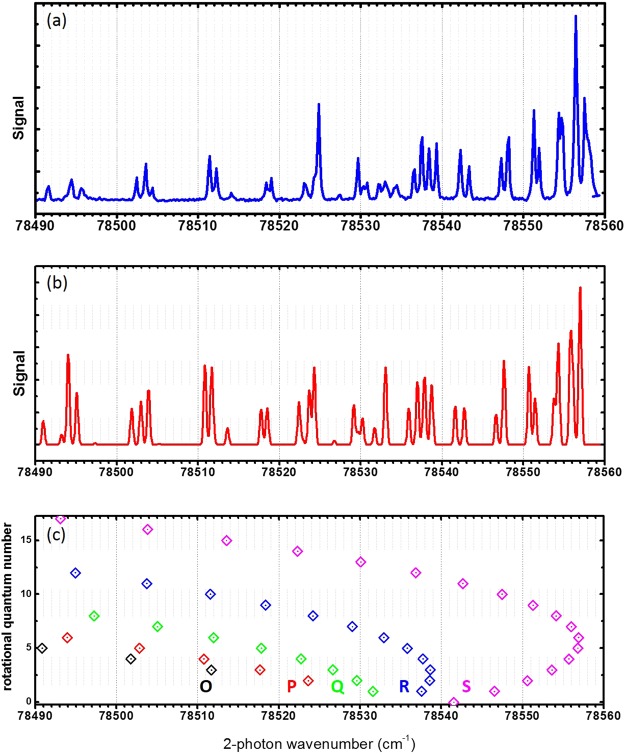
(**a**) Spectrum of the ${a}^{1}{{\rm{\Pi }}}_{g}({\rm{\nu }}=\mathrm{6)}\leftarrow {X}^{1}{{\rm{\Sigma }}}_{g}^{+}({\rm{\nu }}=\mathrm{0)}$ transition in nitrogen from using 2 + 1′ REMPI, with the final ionisation laser tuned so that the total energy of the photons is above the ionisation threshold. (**b**) The PGOPHER simulation at a temperature of 300 K. The full spectrum; both line positions and peak heights, closely match the experimental data. (**c**) The transition designations for each of the branches. The y-axis shows the rotational number for the initial state in ${X}^{1}{{\rm{\Sigma }}}_{g}^{+}({\rm{\nu }}=\mathrm{0)}$.

The 2 + 1 single colour REMPI scheme via the ${a}^{1}{{\rm{\Pi }}}_{g}({\rm{\nu }}=\mathrm{10)}$ state was first investigated in 1989 by Opitz *et al*. to generate ${{\rm{N}}}_{2}^{+}$ ions state selectively^27^. With the intermediate state being the lowest electronic singlet state the 2 + 1 REMPI at 42190 cm^−1^ allows the highly selective preparation of nitrogen ions in specific vibrational states without significant contributions from higher order REMPI processes. However, the rotational state of the ion is only constrained by propensity rules rather than firm selection rules, because the outgoing electron can leave with variable angular momentum. In addition, the process is inefficient due to the small Frank-Condon factor of the transition. In order to overcome these limitations we employ the same electronic ${a}^{1}{{\rm{\Pi }}}_{g}$ state but target a lower vibrational excitation. Changing the intermediate vibrational state from (*v* = 10) to (*v* = 6) increases the Frank-Condon factor by around one order of magnitude^32^ leading to an expected increased efficiency for generating ions also by one order of magnitude^33^. In addition, the resonant transition wavenumber is decreased to 39275 cm^−1^, inhibiting the 2 + 1 single colour transition. We also use a two colour excitation to attain near threshold ionisation and therefore to constrain the rotational states on energy grounds. Figure 1 shows the REMPI spectrum with the two-photon laser being scanned over a wavelength range of 70 cm^−1^ and the ionisation laser set to a wavenumber of 47173 cm^−1^, well above the ionisation threshold to ensure a high efficiency. The spectrum is fitted with the software PGOPHER^34^ and yields rotational constants of (1.99151 ± 0.00292) cm^−1^ and (1.50035 ± 0.00229) cm^−1^ for the ${X}^{1}{{\rm{\Sigma }}}_{g}^{+}({\rm{\nu }}=\mathrm{0)}$ and ${a}^{1}{{\rm{\Pi }}}_{g}({\rm{\nu }}=\mathrm{6)}$ states respectively, which are consistent with the literature values^32,35^. The calibrated transition energy was found to deviate by (2.79 ± 0.04) cm^−1^ from the accepted value of 78530.306 cm^−1^. The peak height distribution corresponds to an internal rotational temperature of the nitrogen molecules of 300 K. This matches the room temperature of the laboratory as expected for a non-supersonic beam expansion. The positions of the peaks are measured with an error of better than 0.01 cm^−1^.

### Spectroscopy of ionisation stage

While the resonant excitation step in the 2 + 1′ REMPI scheme can provide some limitation of possible rotational states in the molecular ion due to propensity rules, the ionisation into one specific ro-vibrational state with high selectivity is not possible. In order to achieve high state selectivity, the ionisation laser needs to be tuned to the ionisation threshold for the specific target state. Similarly to Mackenzie *et al*.^26^, the ionisation threshold for the 2 + 1′ REMPI process is determined by tuning the excitation laser to the desired intermediate state and scanning the ionisation laser while observing the signal.

**Figure 2:**
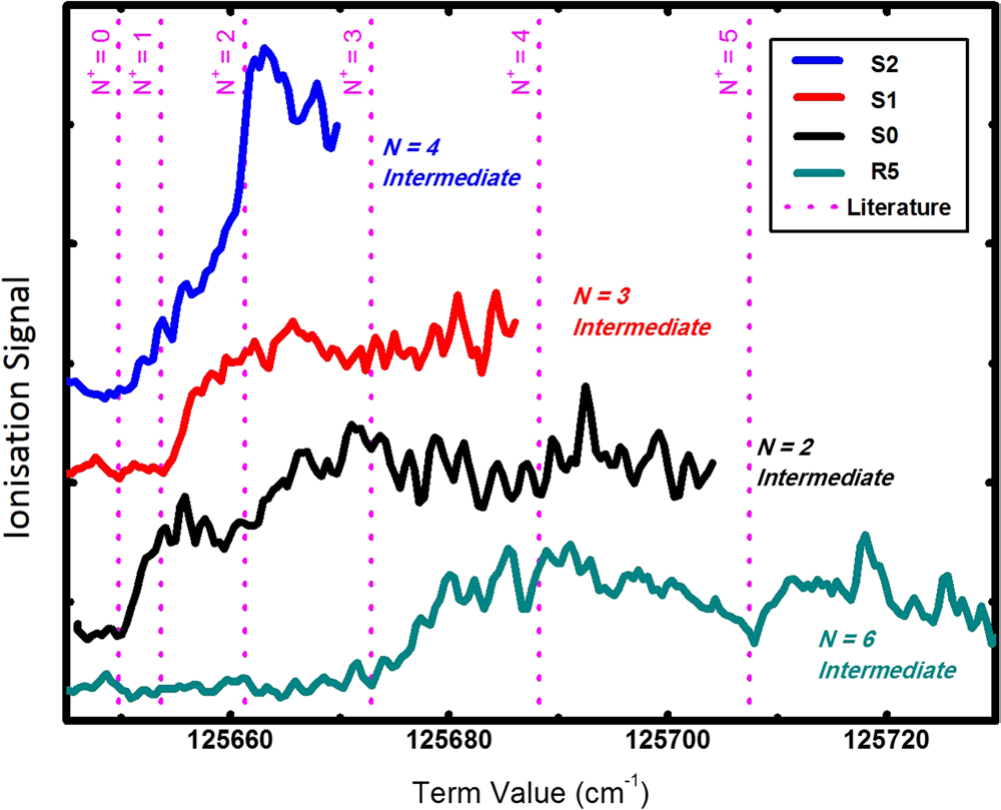
Four scans of the ionisation laser when the excitation laser is set to be resonant with different transitions. Each of the transitions were selected as they were well separated in the excitation spectra (Figure 1), and each goes via a different intermediate rotational state. The baselines of all spectra overlap and are vertically shifted for clarity. The excitation transitions used are S(0), S(1), S(2) and R(5), and the corresponding intermediate rotational states for these are indicated on the graph. The ionisation thresholds (IT) have been fit to the literature values for the spacing of the rotational states in ${X}^{2}{{\rm{\Sigma }}}_{g}^{+}({\rm{\nu }}=\mathrm{0)}$ in ${{\rm{N}}}_{2}^{+}$, and are marked as pink dotted lines. Using these lines as a guide it is also possible to notice additional onsets for loading into higher rotational states. The x-axis is the term value for each scan, which is the total energy relative to the ground rotational state in ${X}^{1}{{\rm{\Sigma }}}_{g}^{+}({\rm{\nu }}=\mathrm{0)}$ in N_2_. The spectral shift of the onsets with respect to the literature values is due to the applied electric field.

Figure 2 shows the ionisation signal for four different intermediate states. For ionisation wavenumbers lower than the ionisation threshold, there is no measurable signal. At the threshold, as expected, the ionisation signal appears as an onset. The spectrum is shifted by Δ*v* = −21.3 cm^−1^ from the literature value of the ionisation threshold due to the presence of a dc electric field across the ionisation region^26^. The shift matches well with the expected shift given by ${\rm{\Delta }}\nu \approx 6.1\,{{\rm{cm}}}^{-1}\cdot \sqrt{{E}_{dc}}$ with the applied electric field of *E*_*dc*_ = 10 V/cm. The electronic ground state wave functions in the neutral molecule and in the molecular ion are both ${{\rm{\Sigma }}}_{g}^{+}$. This results, together with the nitrogen nuclear spin of one, in an even-odd staggering of the rotational states. For the transitions where the initial ground state of the neutral is an even-numbered rotational state (as in the S(0) or S(2) transitions to the intermediate), nuclear spin conservation throughout both steps of the 2 + 1′ process requires that only even rotational levels of the ion (N^+^ = 0, 2 etc) can be produced in the REMPI process, hence the onset occurs at the N^+^ = 0 threshold. Correspondingly for initial odd numbered states (S(1), R(5) transitions), only onsets at the thresholds associated with odd numbered ionic rotational levels (N^+^ = 1, 3) are observed. Thus for the S(0) transition, when the ionisation laser energy is tuned to lie between the N^+^ = 0 and N^+^ = 2 thresholds, only ions in the rovibronic ground state with N^+^ = 0 are produced - only N^+^ = 0 ions are energetically possible even if the excitation is to an autoionised N^+^ = 2 Rydberg state. At energies above the N^+^ = 2 threshold, both N^+^ = 0 and N^+^ = 2 states are likely to be produced, hence the ionisation is no longer a fully state selective process. Likewise for the S(1) transition only ions in the N^+^ = 1 state will be produced at ionisation energies between the N^+^ = 1 and 3 thresholds, but a mix of states will be produced above the N^+^ = 3 threshold.

The detailed structure observed in the traces in Figure 2 is attributable to the contribution from autoionising Rydberg states converging to higher rovibronic thresholds.

## Discussion

**Figure 3:**
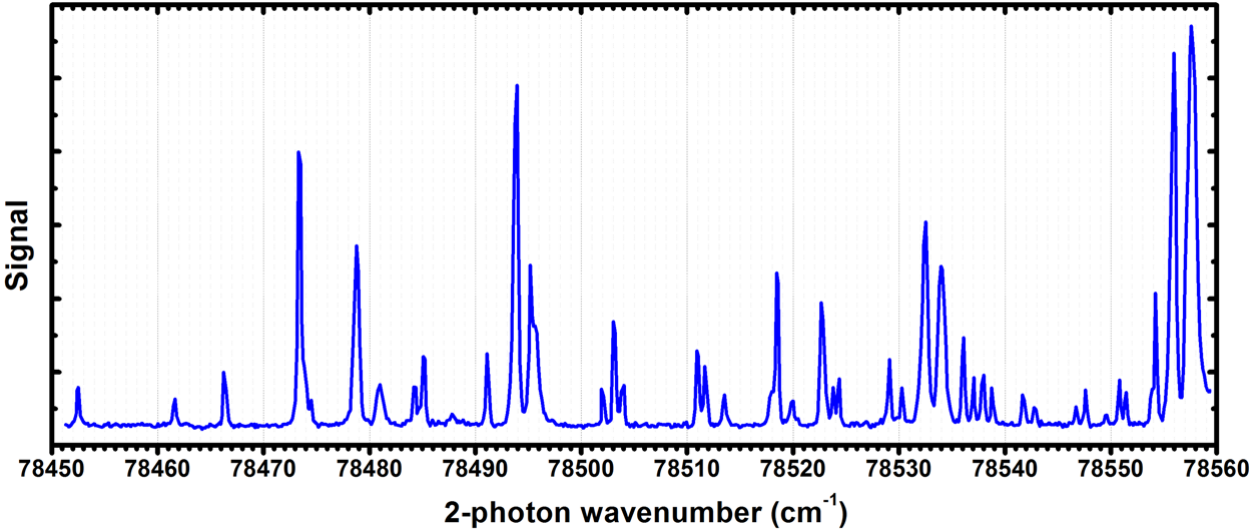
Spectrum taken with just the excitation laser on, resulting in 2 + 2 REMPI. The peak positions are the same as for the 2 + 1′ REMPI spectrum shown in Figure 1, although there is some difference to the relative peak heights.

**Figure 4:**
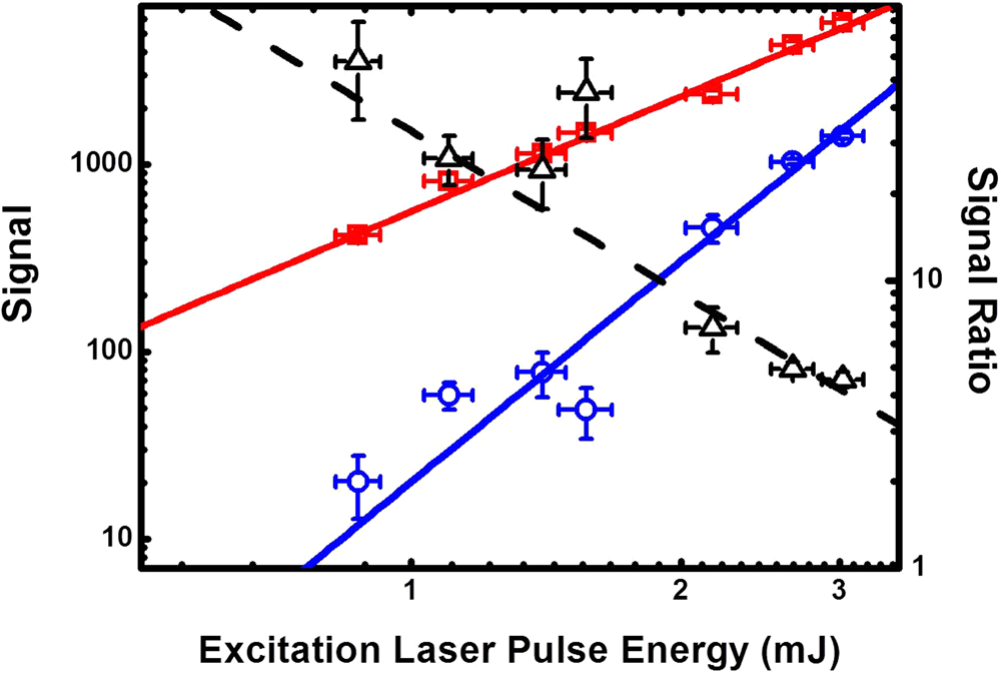
The variation of the signal with excitation laser pulse energy, for the 2 + 2 REMPI (in blue) and for the 2 + 1′ REMPI process (in red). Also shown is the ratio between the two signals (black). The results have been fit with a power law (solid lines) which confirm the quadratic and quartic behaviour of the 2 + 1′ and the 2 + 2 REMPI processes respectively. At low laser pulse energies, the 2 + 1′ REMPI signal dominates and the 2 + 2 signal contributes only 1% at 1 mJ whereas for high laser energies, this contribution increases to 23% for a pulse energy of 3 mJ.

In order to determine how state selective the 2 + 1′ REMPI process is we have analysed contributions to the ionisation through other processes. In the two-photon excitation process, off-resonant excitation of neighbouring transitions can lead to population in unwanted rotational states in the intermediate level. This contribution depends strongly on the density of the transitions in the vicinity of the target transition. For efficiently generating a nitrogen ion in its rotational ground state the O(4), P(2), P(4), R(0), R(2), S(0) and S(2) transitions can be used. The possible R-branch transitions overlap rendering them unsuitable (see Figure 1(c)). The P(2) has a significant overlap with the R(8) transition. Even though, for a cold molecular beam, the contribution from the R(8) transition to the ionisation process can be small, at room temperature, its contribution to the ion generation is well above 10% for the spectroscopic resolution of our system of 0.3 cm^−1^. The other suitable transitions in the spectrum are well separated from their nearest neighbours, with a maximum spectral overlap of 10^−4^. However, the actual contribution to the REMPI signal depends strongly on the neutral molecules’ internal temperature and the spectral broadening of the employed system. The second contribution to the infidelity of the state selectivity is the ionisation stage. Due to the presence of the electric extraction field, Rydberg states which are excited during the laser pulses can be field ionised, leading to a significant shift and broadening of the ionisation signal onset. This effect can be eliminated by removing the electric field during the REMPI process as demonstrated by S.R. Mackenzie *et al*.^26^. The broadening of the onset, however, does not result in a reduction of the state-selectivity as long as the laser is tuned in the region between the field-shifted N^+^ = 0 threshold and the field-shifted N^+^ = 2 threshold because only the production of the state is energetically possible. The last contribution comes from higher order REMPI processes. There is no measurable ionisation of nitrogen molecules with only the ionisation laser. This was confirmed by scanning the ionisation laser wavelength at its highest laser fluence over the range used in Figure 2. In contrast to this, using only the excitation laser shows a clear 2 + 2 REMPI signal. At high laser fluence, a clear excitation spectrum can be seen when the laser wavelength is scanned (see Figure 3). To evaluate its contribution to the ionisation signal, the 2 + 1′ as well as the 2 + 2 REMPI signals are measured for a range of laser fluences. For the 2 + 1′ REMPI measurement, the ionisation laser is set to a fixed laser energy of (2.15 ± 0.09) mJ and the excitation laser is tuned to the S(8) transition. To obtain the 2 + 2 REMPI signal the ionisation laser is switched off. The results are shown in Figure 4. As expected, the REMPI signal for the 2 + 1′ REMPI follows a quadratic increase with increasing excitation laser pulse energy. The 2 + 2 REMPI signal, however, increases quartically. Figure 4 also shows the ratio between the two signals. For low laser pulse energies, the 2 + 1′ REMPI signal dominates and the 2 + 2 signal contributes only 1% at 1 mJ whereas for excitation laser pulse energies of 3 mJ this contribution increases to 23%. Compared to the previous contributions to the infidelity of the state selective ionisation process, the 2 + 2 REMPI dominates the production of non-state selected molecular ions.

In conclusion, we have investigated a novel highly state-selective REMPI scheme to generate nitrogen ions in the ro-vibrational ground state. Using a two-colour REMPI via the ${a}^{1}{{\rm{\Pi }}}_{g}({\rm{\nu }}=\mathrm{6)}$ intermediate state a state selectivity of better than 99% can be achieved for generating ${{\rm{N}}}_{2}^{+}$ ions in their ro-vibrational ground state. The high state selectivity is reached by energetically inhibiting the generation of ions in higher rotational states. Even though this approach provides high state selectivity of the rotational ground state, higher rotational states can only be generated as a mixture of states with its composition governed by propensity rules (similar to^26^). Given that the ${{\rm{N}}}_{2}^{+}$ ion is non-polar, the lifetime of the rotational state is practically only limited by collisions which leads to fast re-thermalisation of the state distribution. As a consequence, great care must be taken to avoid intra-beam collisions during and after the REMPI process^31^.

## Methods

To investigate the relative ionisation efficiency, we employ a time-of-flight (TOF) mass spectrometer. A pulsed molecular beam is generated by injecting gas into a high vacuum system through a solenoid valve (Parker Series 8 valve) with an orifice diameter of 0.52 mm and a stagnation pressure of 1 bar. The pulse length is set to 139 *μ*s in order to avoid excessive increase of the background pressure in the vacuum system. The molecular beam passes through the TOF spectrometer where the molecules interact with the two REMPI lasers. The remaining neutral molecules are removed with an Oerlikon Leybold TURBOVAC 361 Turbo pump, which has a pumping speed of 400 l/s for N_2_. In order to keep the background pressure below 5 × 10^−6^ mbar, only 40 gas pulses are generated in a 2 Hz repetition rate sequence which is followed by 10 s pump-down time.

**Figure 5:**
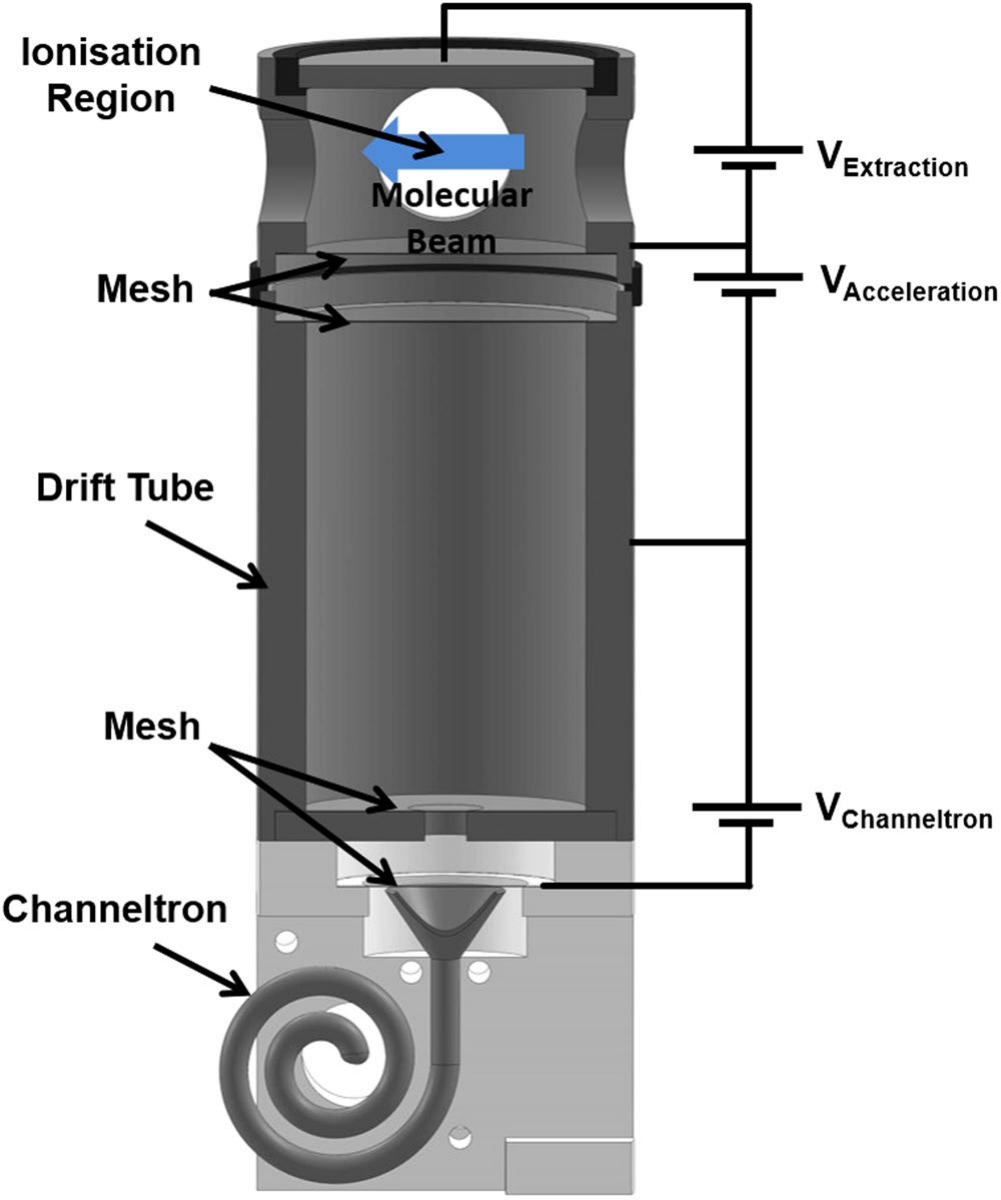
The time-of-flight (TOF) mass spectrometer for detection of ${{\rm{N}}}_{2}^{+}$. In the ionisation region the laser beams and nitrogen beam (blue arrow) cross orthogonally. Voltages are applied to the capacitor plate, the extraction box, the drift tube, and the cone of the channel electron amplifier so as to maximise the ${{\rm{N}}}_{2}^{+}$ signal, as well as separating it in the TOF spectrum from other artifacts.

To detect the ions generated in the molecule-laser interaction, a compact TOF system is employed (see Figure 5), with the distance between the extraction plate and the first mesh being 22 mm. The positive voltage (*V*_*Extraction*_) on the extraction electrodes at the end of the TOF setup injects the ions into the acceleration region through a metal mesh. After the ions are accelerated by the potential difference *V*_*Acceleration*_ the ions enter the drift tube through another metal mesh. The ions are then detected by accelerating them with a 2.5 kV potential difference between the end of the drift tube and the entrance of the channel electron multiplier. A transimpedance amplifier converts the current signal from the channeltron into a voltage signal which is then measured with an oscilloscope. The oscilloscope trace is loaded into a computer and the TOF peak is integrated and summed over all the laser pulses.

Two frequency-doubled dye lasers (Sirah Cobra Stretch and Radiant Dyes Narrowscan lasers) are used, each of which is pumped with the third harmonic of one of two Continuum Nd:YAG lasers (Continuum Surelite II @ 10 Hz). The Radiant Dyes laser is used for the excitation stage at 39270 cm^−1^ (255 nm) (Coumarin 307 in Ethanol), and the Sirah Cobra laser is used for the ionisation stage at 47110 cm^−1^ (212 nm) (Stilbene 3 in Ethanol). Typical pulse energies used for the experiment are in the range of 1 mJ to 5 mJ. The wavelengths of the lasers are measured throughout the experiment with a wavelength meter (Highfinesse WS7) with an absolute accuracy of 60 MHz.

In order to adjust the timing between the gas pulses and the two REMPI laser pulses, two delay generators are used. The first generator triggers the flash lamps of the two pump lasers and it triggers the second delay generator. The second delay generator in turn triggers the pulsed valve as well as the Q-switches of both pump lasers. Adjusting the delay between the flash lamp trigger and the Q-switch is used to optimise the laser pulse timing and the laser fluence. In addition, the delay is used to optimise the temporal overlap between the gas pulse and the laser pulse in the interaction region of the TOF.

### Characterisation of the Setup

**Figure 6:**
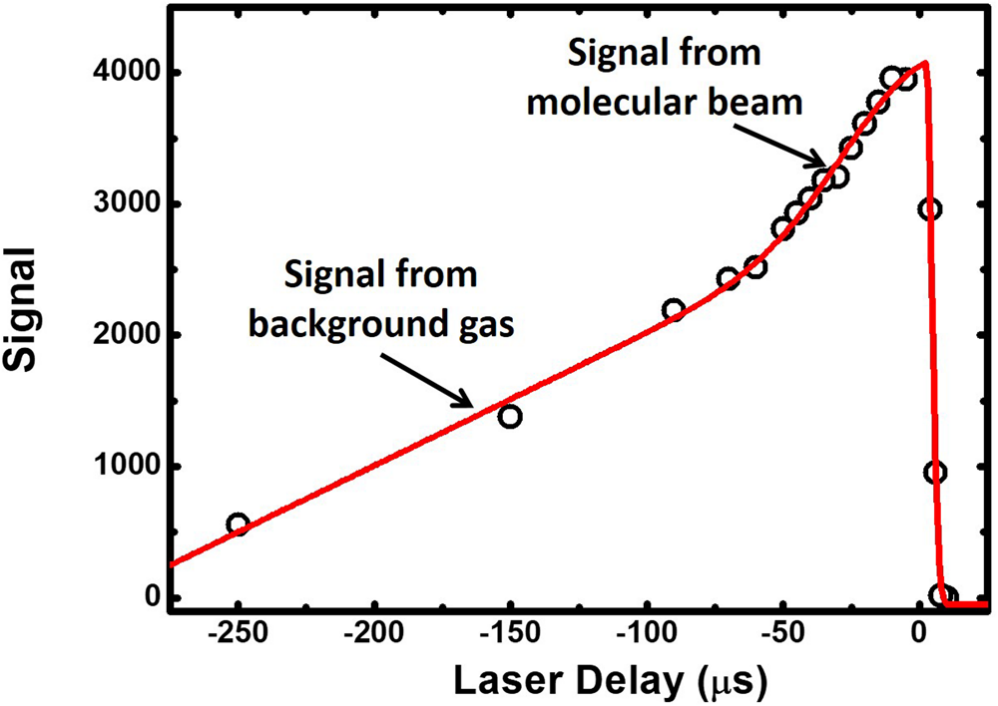
The change in signal as the timing of the excitation laser is scanned for fixed timing of the ionisation laser (at the peak of its interaction with the gas pulse). When the excitation laser arrives after the ionisation laser, there is a sharp drop off in signal. If the laser timing is such that the excitation laser arrives first, there is a much slower reduction in signal with time delay, due to diffusion of excited molecules through the laser beams.

Initially, the system is optimised by observing the TOF ion signal when the lasers are tuned on resonance with the intermediate state. The lasers are overlapped so that they are counter-propagating through the spectrometer, and each one is focused into the centre of the extraction region using lenses with focal lengths 300 mm. Their spatial overlap is optimised by maximising the REMPI signal from the TOF spectrometer. The temporal laser pulse overlap is coarsely adjusted by measuring the arrival times of the pulses with a fast photo diode. The fine tuning of the overlap is achieved by observing the TOF signal for various time delays between the laser pulses (see Figure 6). The plot of the signal vs the laser pulse delay shows a steep onset of the signal when the two laser pulses are overlapped followed by a steady decrease of signal as the molecules excited in the two photon transition by the first laser pulse move out of the laser beam before the ionisation laser. The slower decrease for longer delays (>50 *μ*s) is a result of the diffusion of excited molecules from the background out of the ionisation laser beam across the length of the beam. Due to the compactness of the TOF spectrometer and the lack of beam skimming there is significant scattering of the molecular beam in the TOF ionisation region leading to a significantly increased local pressure. This results in an intra-molecular temperature of about 300 K.

**Figure 7:**
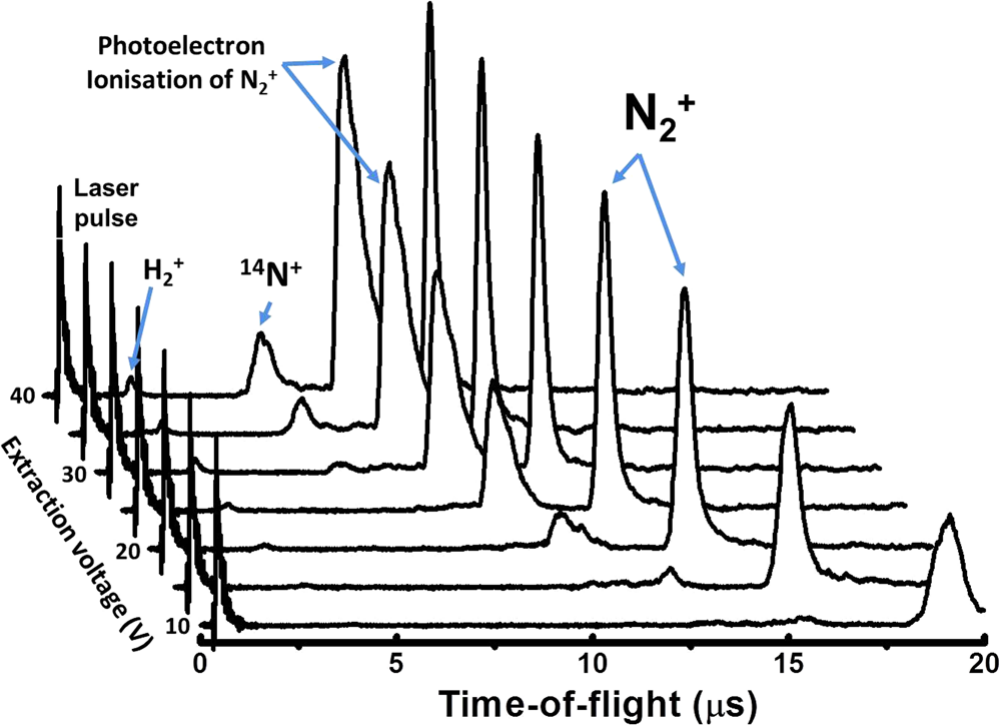
The time of flight (TOF) profile for a series of extraction voltages, with the highest voltage applied being 40 V (background) and the lowest voltage of 10 V (foreground), with 5 V steps between each trace. Visible are signatures indicating the laser arrival, H_2_, Nitrogen atoms, and finally the resonant ${{\rm{N}}}_{2}^{+}$ peak over which the signal is integrated.

In addition to the spatio-temporal laser pulse overlap, the operation voltages of the TOF need to be optimised to clearly distinguish the REMPI signal from background signal generated by the laser ionisation of molecules in the background gas as well as atomic and molecular ions generated by photo-electron ionisation of molecules in the beam and background gas. Measuring the TOF arrival time distribution allows identification of the different contributions to the signal (see Figure 7). The pump laser Q-switch pulse creates an electronic interference signal indicated in the TOF profiles. This sharp peak is followed by a small signal created by the ionisation of the background hydrogen in the system. The peak following the hydrogen signal is nitrogen atoms created by the dissociation of molecular nitrogen. The largest integrated peak for high extraction voltages is due to molecular ions generated by photoelectron impact ionisation of the gas pulse. Photoelectrons generated by the laser pulses are accelerated by the extraction field towards the repellor electrode where nitrogen is impact ionised. The height of the peak decreases with decreasing extraction voltage as the ionisation cross section of electron impact ionisation diminishes towards the ionisation threshold. Even though the ions are created close to the repellor electrode on top of the TOF spectrometer, the signal arrives before the REMPI signal because the ions are accelerated in the extraction field to higher velocities and therefore overtake ions generated in the centre of the excitation region via REMPI in the drift tube. The height of this peak is independent of the laser wavelength and is present independent of which REMPI laser is applied. The peak disappears when the pulsed valve is not opened. This confirms the origin of this peak. The final peak in the TOF is the resonant ${{\rm{N}}}_{2}^{+}$ peak. The window for integrating this signal was chosen to be 2 *μ*s across the peak and the extraction voltage set to 20 V, which is a good compromise between separating the REMPI signal from the other contributions in the TOF output and still having a peak height well above the electronic noise level of the detection system.

In order to determine the inhomogeneity of the electric field, we analysed the width of the REMPI signal. With laser beam diameters on the order of 50 *μ*m an arrival time broadening of less than 100 ns is expected and the thermal velocity distribution at 300 K results in a width of ≈220 ns. We therefore attribute the remaining broadening solely to the inhomogeneity of the electric extraction field, which results in a field inhomogeneity of 0.64 V/cm. The resulting broadening of the ionisation stage spectrum due to the field-induced shift is therefore ≈4.8 cm^−1^.
